# Unlabeled Insight, Labeled Boost: Contrastive Learning and Class-Adaptive Pseudo-Labeling for Semi-Supervised Medical Image Classification

**DOI:** 10.3390/e27101015

**Published:** 2025-09-27

**Authors:** Jing Yang, Mingliang Chen, Qinhao Jia, Shuxian Liu

**Affiliations:** School of Computer Science and Technology, Xinjiang University, Urumqi 830049, China; 107552304184@stu.xju.edu.cn (J.Y.); 107552304033@stu.xju.edu.cn (M.C.); 107552304069@stu.xju.edu.cn (Q.J.)

**Keywords:** semi-supervised learning, data imbalance, medical image classification, contrastive learning, computer vision

## Abstract

The medical imaging domain frequently encounters the dual challenges of annotation scarcity and class imbalance. A critical issue lies in effectively extracting information from limited labeled data while mitigating the dominance of head classes. The existing approaches often overlook in-depth modeling of sample relationships in low-dimensional spaces, while rigid or suboptimal dynamic thresholding strategies in pseudo-label generation are susceptible to noisy label interference, leading to cumulative bias amplification during the early training phases. To address these issues, we propose a semi-supervised medical image classification framework combining labeled data-contrastive learning with class-adaptive pseudo-labeling (CLCP-MT), comprising two key components: the semantic discrimination enhancement (SDE) module and the class-adaptive pseudo-label refinement (CAPR) module. The former incorporates supervised contrastive learning on limited labeled data to fully exploit discriminative information in latent structural spaces, thereby significantly amplifying the value of sparse annotations. The latter dynamically calibrates pseudo-label confidence thresholds according to real-time learning progress across different classes, effectively reducing head-class dominance while enhancing tail-class recognition performance. These synergistic modules collectively achieve breakthroughs in both information utilization efficiency and model robustness, demonstrating superior performance in class-imbalanced scenarios. Extensive experiments on the ISIC2018 skin lesion dataset and Chest X-ray14 thoracic disease dataset validate CLCP-MT’s efficacy. With only 20% labeled and 80% unlabeled data, our framework achieves a 10.38% F1-score improvement on ISIC2018 and a 2.64% AUC increase on Chest X-ray14 compared to the baselines, confirming its effectiveness and superiority under annotation-deficient and class-imbalanced conditions.

## 1. Introduction

Traditional machine learning algorithms, such as SVMs, Random Forests, and KNNs, perform well on small-scale datasets. These methods established the foundation for early medical image analysis [1,2,3]. Deep learning models, such as CNNs and Transformers, leverage large annotated datasets and high-performance computing. They overcome the limitations of traditional machine learning in feature extraction and pattern recognition. These models achieve automated and high-precision classification of large-scale medical imaging data [4,5,6].

The widespread adoption of deep learning across various domains has significantly accelerated industrial advancements. Medical image classification is a core component of medical image analysis. Deep learning models significantly reduce the workload of radiologists in both training and clinical practice while providing reliable auxiliary data for downstream diagnosis [7]. However, training deep learning models requires extensive annotated data support. Medical data annotation requires expert knowledge and careful review. As a result, the annotation costs are much higher than in other image domains [8]. In recent years, semi-supervised learning, with its significantly reduced reliance on labeled data compared to traditional supervised learning, has gained widespread application across various deep learning tasks [9,10]. Semi-supervised learning splits datasets into a small labeled subset and a large unlabeled subset. It uses strategies such as pseudo-labeling [11,12], consistency regularization [13,14], and label propagation [15] to train deep models with limited labeled data. This approach dramatically reduces annotation costs, and numerous studies [16,17,18] have successfully introduced semi-supervised learning into medical image classification tasks.

In the current semi-supervised image classification methodologies, two predominant approaches have emerged [19,20]: consistency-based methods, which enforce prediction stability under input perturbations, and pseudo-labeling-based methods, which assign pseudo-labels to unlabeled data before retraining with the augmented set.

Among them, consistency-based methods enforce prediction stability under input perturbations, as exemplified by the mean teacher (MT) framework [21], which aligns student and teacher model predictions. Extensions of this line of work have further incorporated relation modeling [17] and contrastive self-supervised pretraining [22] to better leverage unlabeled data. Pseudo-labeling approaches, on the other hand, generate supervisory signals from model predictions to expand the training set [23,24]. Pseudo-labeling approaches have drawn increasing attention due to their simplicity and scalability. Early works such as FixMatch [25] employed fixed-threshold pseudo-labeling but discarded a large portion of unlabeled data, particularly those from minority classes. FlexMatch [26] introduced dynamic per-class thresholds but remained ineffective under class imbalance. Recent studies use adaptive thresholds [27], label smoothing [28], curriculum learning [29], or distribution modeling [30]. These techniques aim to reduce imbalance and improve pseudo-label reliability. Other works leveraged prototype alignment [31] or multi-level feature fusion [23]. Despite these advances, two major limitations remain regarding the existing approaches: (i) they exhibit simplistic utilization patterns of labeled data, neglecting the modeling of intermediate feature layers and multi-scale structural information; (ii) they employ pseudo-label threshold adjustment mechanisms with inherent limitations that accumulate bias during the initial training phases due to noisy labels.

To address these challenges, this paper proposes a novel semi-supervised medical image classification framework integrating contrastive learning with category-adaptive pseudo-labeling. We design a semantic discrimination enhancement module that strengthens the utilization of labeled data through supervised contrastive loss, thereby improving feature representation by reducing intra-class distances while increasing inter-class separation. This ensures reliable model convergence during the early training stages when labeled data is scarce and learnable information limited. Considering the inherent class imbalance in medical imaging, where models tend to favor majority classes, we develop a category-adaptive pseudo-label regulation module that dynamically adjusts thresholds based on per-class learning progress, effectively alleviating head-class dominance while improving tail-class recognition. To minimize early-stage noise interference from erroneous pseudo-labels when model capability is weak, our method strategically implements category-adaptive pseudo-labeling in the later training phases. Furthermore, we exploit deep semantics in unlabeled data by enforcing consistency across different views of the same sample. Dual augmented views and additional regularization improve robustness to semantic features. This leads to better classification performance. Extensive experiments on the ISIC2018 and Chest X-ray14 datasets demonstrate significant improvements in classification accuracy. The main contributions of this work are summarized as follows:

- We propose a novel semi-supervised medical image classification model, CLCP-MT, which addresses the dual challenges of annotation scarcity and class imbalance by integrating supervised contrastive learning with a category-adaptive pseudo-labeling mechanism, thereby significantly enhancing overall classification performance.
- We design a semantic discrimination enhancement (SDE) module that leverages supervised contrastive learning to cluster intra-class samples while separating inter-class samples, effectively extracting discriminative information from limited labeled data in latent structural space and substantially amplifying the value of sparse annotations.
- We introduce a category-adaptive pseudo-label regulation (CAPR) module, which dynamically adjusts pseudo-label confidence thresholds based on real-time learning progress across different categories, mitigating dominance by head classes while improving recognition performance for tail classes, thereby achieving effective modeling of long-tailed distributions.
- The experimental results on the ISIC2018 and Chest X-ray14 datasets demonstrate that our method consistently outperforms existing semi-supervised approaches under varying annotation ratios and exhibits remarkable efficacy and robustness in class-imbalanced scenarios.

## 2. Related Work

Semi-supervised learning has gained widespread adoption across various computer vision tasks by jointly leveraging a limited set of labeled images alongside abundant unlabeled data during training. The current methodologies can primarily be categorized into two paradigms: pseudo-labeling strategy [11] and consistency regularization [32]. The former generates pseudo-labels for unlabeled data based on model predictions and then uses them for further training, while the latter imposes perturbations at the input, feature, or network levels, encouraging the model to produce consistent outputs across multiple views of the same image, thereby enhancing robustness.

### 2.1. Pseudo-Labeling

**Figure 1:**
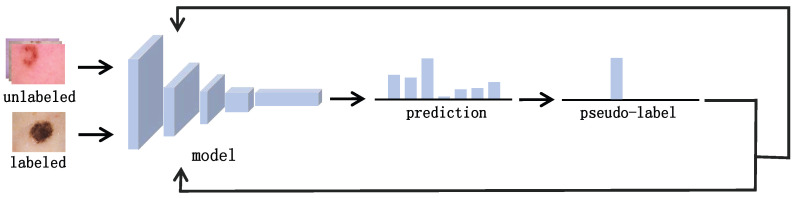
Pseudo-labeling method diagram.

The core concept of the pseudo-labeling method in classification tasks is as follows: Initially, a base classification model is trained using existing labeled data. This model then predicts labels for unlabeled data, with high-confidence predictions selected as pseudo-labels. After screening, these pseudo-labels are combined with the original labeled data for subsequent model retraining, as illustrated in Figure 1. This approach effectively leverages the latent information within unlabeled data, demonstrating remarkable performance in mitigating insufficient labeled samples and enhancing model generalization capabilities, thus being widely adopted in semi-supervised classification tasks. However, this method is highly sensitive to pseudo-label accuracy. Biases in the initial model may introduce erroneous labels during training, potentially compromising the final performance. Consequently, the research on pseudo-labeling primarily focuses on improving pseudo-label reliability and designing robust confidence-based selection mechanisms to ensure stable and effective learning of useful information from unlabeled data. For instance, Sohn et al. [25] proposed the FixMatch model, which generates pseudo-labels for unlabeled data meeting a fixed confidence threshold. Zhang et al. [26] introduced the FlexMatch model, dynamically adjusting class-specific thresholds over time. Peng et al. [27] developed FullMatch, incorporating adaptive thresholding to generate more pseudo-labels for challenging classes. Zhou et al. [28] proposed FixMatch-LS, integrating label smoothing to mitigate the impact of erroneous pseudo-labels. Peng et al. [29] presented FaxMatch, combining label smoothing for soft pseudo-labels with curriculum learning and dynamic thresholds. Zhou et al. [33] introduced the growth threshold for pseudo-labeling (GTPL), which adjusts class-specific thresholds by integrating confidence scores from both labeled and unlabeled data.

### 2.2. Consistency Regularization

The core concept of consistency regularization lies in minimizing the discrepancy between outputs generated from differently perturbed versions of unlabeled data. This approach significantly enhances model robustness and generalization capability without incurring additional annotation costs. However, it exhibits high sensitivity to perturbation design: excessive perturbations may violate the “semantic invariance” assumption, while insufficient perturbations fail to provide meaningful constraints. Furthermore, error accumulation may occur due to amplified incorrect consistency stemming from mean teacher architectures, pseudo-labeling biases, or inherent network biases. Establishing high-quality consistency targets during training is crucial for optimal performance, making this method widely adopted. For instance, Tarvainen et al. [21] proposed the mean teacher (MT) model, which constructs the teacher model using exponential moving average (EMA) values of student model parameters and enforces prediction consistency between them. Liu et al. [17] developed the SRC-MT model, incorporating consistency regularization to model inter-sample relationships. Guo et al. [34] introduced CamMix, a mixed-sample data augmentation method based on consistency regularization that effectively leverages sample relationships. Hang et al. [35] proposed RAC-MT, which employs dynamic weighting to selectively utilize reliable unlabeled data, addressing reliability concerns in unlabeled datasets. Gai et al. [24] presented a semi-supervised medical image classification method featuring class-prototype matching and soft pseudo-label consistency regularization, utilizing a prototype matching module for soft pseudo-label prediction and a linear mixing strategy for both labeled and unlabeled data to improve classification performance.

## 3. Methods

**Figure 2:**
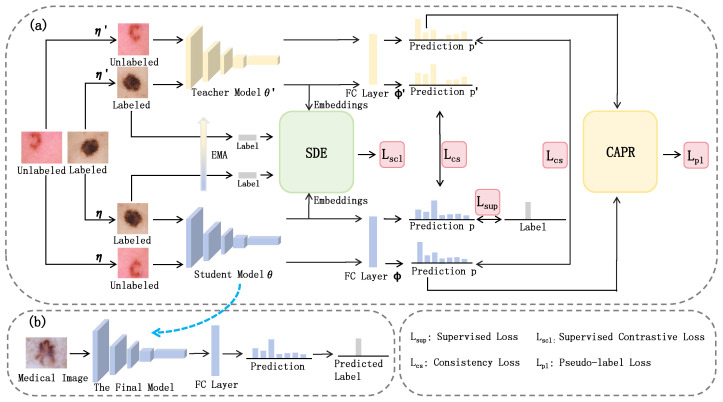
Overview of the proposed CLCP-MT framework. (**a**) The training process, wherein student model and teacher model are two parallel networks with the same architecture but different initializations. The CAPR module is incorporated in the later stages to mitigate the impact of erroneous pseudo-labels. (**b**) The classification procedure post-training, with the final model being determined by the student network.

This section delineates the semi-supervised medical image classification model CLCP-MT proposed in our study, as illustrated in Figure 2. The overall architecture builds upon the mean teacher framework [21], incorporating the consistency regularization principle: applying diverse perturbations to identical inputs while constraining output-level consistency to fully exploit latent information from unlabeled data. Within the mean teacher paradigm, the teacher model parameters $\theta^{\prime }$ are updated via exponential moving average (EMA) of student model parameters θ, with gradient optimization exclusively applied to the student network during training. This approach leverages temporally ensembled student weights to construct a more robust teacher network for generating reliable consistency targets to guide student model training.

To effectively extract discriminative information under extreme label scarcity, we augment this framework with a semantic discriminative enhancement (SDE) module, introducing supervised contrastive loss to optimize utilization of limited labeled samples. Furthermore, addressing the challenges of error amplification from noisy pseudo-labels during the early training phases and head-class dominance, we implement a class-adaptive pseudo-label refinement (CAPR) module in the later training stages. This component employs dynamic thresholding to suppress head-class bias while enhancing tail-class recognition, thereby improving overall model performance on class-imbalanced medical datasets.

### 3.1. Semantic Discrimination Enhancement (SDE) Module

**Figure 3:**
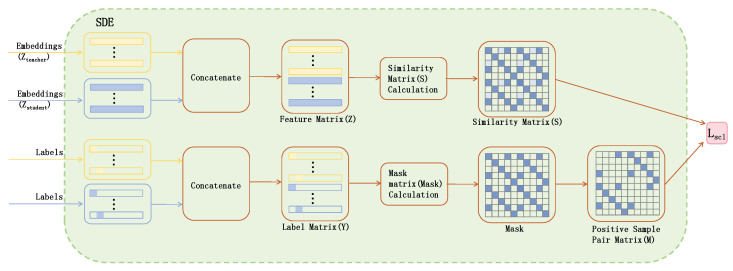
Semantic discrimination enhancement module (SDE).

To fully exploit the informational value of labeled data and deeply investigate the relationships among data in low-dimensional space, we designed a semantic discrimination enhancement (SDE) module, as illustrated in Figure 3. By employing contrastive loss on labeled data, our approach minimizes intra-class distances while maximizing inter-class distances within the labeled dataset, thereby encouraging the network to extract additional semantic information from the scarce labeled samples.

The labeled data is subjected to two distinct perturbations, η and $\eta^{\prime }$, before being fed into the student network and teacher network, respectively, yielding embeddings $Z_{student}$ and $Z_{teacher}$. These embeddings can be expressed as

(1) $$Z_{student}={\left[z_{1},z_{2},\ldots ,z_{N}\right]}^{⊤}$$

(2) $$Z_{teacher}={\left[z_{1}^{\prime },z_{2}^{\prime },\ldots ,z_{N}^{\prime }\right]}^{⊤}$$

where $z_{i}$ and $z_{i}^{\prime }$ represent the embeddings obtained from the student network and the teacher network, respectively, after inputting the i-th image with different perturbations. We concatenate $Z_{student}$ and $Z_{teacher}$ along dimension 0 to obtain the feature matrix *Z*, which can be expressed as

(3) $$Z={\left[z_{1},\ldots ,z_{N},z_{1}^{\prime },\ldots ,z_{N}^{\prime }\right]}^{⊤}$$

For clarity in notation, we denote *Z* as

(4) $$Z={\left[z_{1},\ldots ,z_{N},z_{N+1},\ldots ,z_{2N}\right]}^{⊤}$$

Since both $Z_{student}$ and $Z_{teacher}$ are derived from the same labeled dataset, the resulting label matrix *Y* obtained by concatenating their corresponding ground truth labels *y* along dimension 0 can be expressed as

(5) $$Y={\left[y_{1},\ldots ,y_{N},y_{1},\ldots ,y_{N}\right]}^{⊤}$$

The similarity matrix *S* serves to identify samples that are proximate within the feature space, which can be expressed as

(6) $$S=Z\cdot Z^{⊤}=\left[\begin{array}{cccc} S_{1,1} & S_{1,2} & \ldots & S_{1,2N}\\ S_{2,1} & S_{2,2} & \ldots & S_{2,2N}\\ \vdots & \vdots & \ddots & \vdots \\ S_{2N,1} & S_{2N,2} & \ldots & S_{2N,2N} \end{array}\right],S_{ij}=z_{i}\cdot z_{j}^{⊤}$$

where $S_{ij}$ denotes the similarity between sample i and sample j. The objective is to minimize the distance between samples i and j in the feature space and enhance their similarity when they belong to the same category while maximizing their separation distance and suppressing their similarity when they pertain to distinct categories.

To identify homogeneous samples, we define a mask matrix Mask, as expressed in Equation (7). When $Mask_{ij}=0$, it indicates that the ground truth labels of sample i and sample j have no overlapping components, meaning the samples belong to distinct classes. Conversely, when $Mask_{ij}>0$, it signifies that the ground truth labels of sample i and sample j share overlapping components, indicating the samples are from the same class. To exclude self-comparisons, the mask matrix Mask must be subtracted by an identity matrix *E*, with the positive sample pair mask matrix *M* represented in Equation (8).

(7) $$Mask=Y\cdot Y^{⊤}=\left[\begin{array}{cccc} Mask_{1,1} & Mask_{1,2} & \ldots & Mask_{1,2N}\\ Mask_{2,1} & Mask_{2,2} & \ldots & Mask_{2,2N}\\ \vdots & \vdots & \ddots & \vdots \\ Mask_{2N,1} & Mask_{2N,2} & \ldots & Mask_{2N,2N} \end{array}\right],Mask_{ij}=y_{i}\cdot y_{j}^{⊤}$$

(8) $$M=Mask-E=\left[\begin{array}{cccc} M_{1,1} & M_{1,2} & \ldots & M_{1,2N}\\ M_{2,1} & M_{2,2} & \ldots & M_{2,2N}\\ \vdots & \vdots & \ddots & \vdots \\ M_{2N,1} & M_{2N,2} & \ldots & M_{2N,2N} \end{array}\right],M_{ij}=\left\{\begin{array}{cc} 0, & \mathrm{if}\;i=j\\ y_{i}\cdot y_{j}^{⊤}, & \mathrm{if}\;i\neq j \end{array}\right.$$

To minimize intra-class sample distances while maximizing inter-class sample distances, the supervised contrastive loss function is defined as

(9) $$L_{scl}=-\frac{\tau }{\tau_{0}}\frac{1}{2N}\sum_{i=1}^{2N}\frac{1}{\sum_{j=1}^{2N}M_{ij}}\sum_{j=1}^{2N}M_{ij}log\frac{e^{\frac{S_{ij}}{\tau }}}{\sum_{k=1}^{2N}1\left\{k\neq 1\right\}\cdot e^{\frac{S^{ik}}{\tau }}}$$

where τ and $\tau_{0}$ denote the temperature coefficients.

### 3.2. Class-Adaptive Pseudo-Label Refinement (CAPR) Module

**Figure 4:**
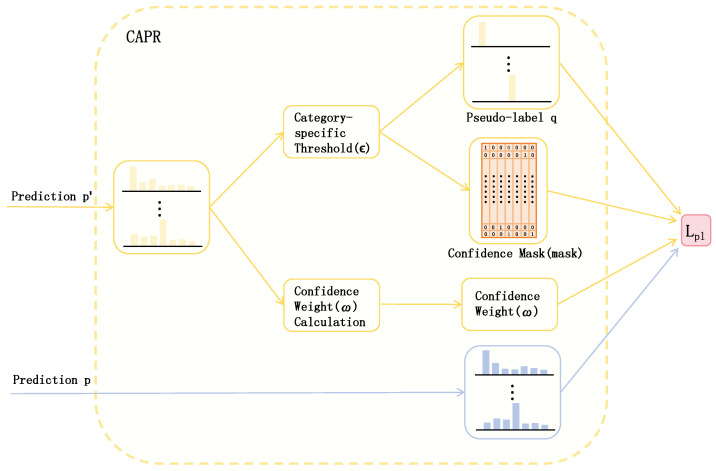
Class-adaptive pseudo-label refinement (CAPR) module.

Medical imaging data often exhibits class imbalance and sample difficulty imbalance. During training, neural networks tend to prioritize learning easily classifiable samples while struggling with the minority of challenging cases. To address this issue, we propose a category-adaptive pseudo-label regulation module that dynamically reduces the weight of easy samples while increasing the weight of hard samples, thereby preventing model training from being dominated by easily classifiable instances, as illustrated in Figure 4.

For each category, a specific threshold is dynamically adjusted for pseudo-label generation. The unlabeled data $x_{i}$ is fed into both the student network and the teacher network after undergoing different perturbations, yielding prediction outputs $p_{i}$ and $p_{i}^{\prime }$, respectively. Based on the category-specific threshold ϵ, the pseudo-label $q_{i}$ corresponding to the teacher network’s prediction $p_{i}^{\prime }$ for the current unlabeled data can be derived. Here, $\epsilon_{c}$ denotes the threshold for the *c*-th category. The computation of the pseudo-label threshold and the pseudo-label for the *c*-th category at the *t*-th epoch is as follows:

(10) $$p_{i}=f\left(x_{i};\theta ,\eta \right)$$

(11) $$p_{i}^{\prime }=f\left(x_{i};\theta^{\prime },\eta^{\prime }\right)$$

(12) $$\epsilon_{c}\left(t\right)=\epsilon_{min}+\left(\epsilon_{max}-\epsilon_{min}\right)\cdot max\left(\frac{t}{E_{ramp}},1\right)\cdot \left[1+0.1\cdot \left(1-v_{c}\right)\right],v_{c}=\frac{1/D_{c}}{\sum_{c=1}^{C}1/D_{c}}$$

(13) $$q_{i}=1\left\{p_{i}^{\prime }>\epsilon_{c}\right\}$$

Herein, f(·) denotes the classification network, where θ and $\theta^{\prime }$ represent the parameters of the student network and teacher network, respectively, η and $\eta^{\prime }$ correspond to the perturbations applied to the student and teacher networks, $\epsilon_{\min}$ signifies the minimum threshold, $\epsilon_{\max}$ indicates the maximum threshold, $E_{\mathrm{ramp}}$ stands for the ramp-up period, $v_{c}$ refers to the class weight, and $D_{c}$ denotes the number of samples belonging to class *c* in the training set.

To mitigate the interference caused by low-confidence pseudo-labels during training, we employ a category-specific threshold ϵ to derive a confidence mask for filtering high-confidence pseudo-label samples, expressed as

(14) $$mask=1\{p_{i}^{\prime }>\epsilon_{c}\vee p_{i}^{\prime }<1-\epsilon_{c}\}$$

In addition to the mask, we quantified the uncertainty of pseudo-labels and weighted the pseudo-label loss based on uncertainty, thereby enabling the model to focus more on high-confidence samples while reducing reliance on low-confidence ones. We employed entropy to measure prediction uncertainty and generated confidence weights based on entropy values. The entropy $H_{i}$ and confidence weight $\omega_{i}$ for unlabeled data are calculated as follows:

(15) $$H_{i}=-\left[p_{i}^{\prime }\cdot logp_{i}^{\prime }+\left(1-p_{i}^{\prime }\right)log\left(1-p_{i}^{\prime }\right)\right]$$

(16) $$\omega_{i}=1-H_{i}$$

By applying the confidence mask and weights $\omega_{i}$, we can compute the pseudo-label loss $L_{pl}$ between the student network’s prediction $p_{i}$ and the teacher network’s generated pseudo-label $q_{i}$ for the same sample. The pseudo-label loss is formulated as follows:

(17) $$L_{pl}=\sum_{i=N}^{N+M}\omega_{i}FL\left(p_{i}\cdot mask,q_{i}\cdot mask\right)=-\omega_{i}\cdot mask\cdot \alpha_{t}{\left(1-p_{t}\right)}^{\gamma }logp_{t}$$

(18) $$p_{t}=\left\{\begin{array}{cc} p_{i}, & \mathrm{if}\;q_{i}=1\\ 1-p_{i}, & \mathrm{otherwise} \end{array}\right.$$

where FL( ) denotes the focal loss function.

### 3.3. Overall Loss

During the initial training phase, the entire batch size (containing both labeled and unlabeled data) is subjected to different perturbations before being fed into the student and teacher models. For the labeled data $x_{i}$(i=1,2,…,N), the predicted results $p_{i}$ are compared with the ground truth labels $y_{i}$ to compute the supervised loss $L_{sup}$, as shown in Equation (19). The consistency loss $L_{cs}$ is derived by comparing the student network’s predictions $p_{i}$ with the teacher network’s predictions $p_{i}^{\prime }$ across the entire batch, as specified in Equation (20). The labeled contrastive loss $L_{scl}$ is obtained by concatenating the feature representations of differently perturbed labeled data after passing through the student and teacher networks, then computing the loss between these data-level features and their corresponding ground truth labels, as illustrated in Equation (9).

(19) $$L_{sup}=\sum_{i=1}^{N}H\left(p_{i},y_{i}\right),H\left(p_{i},y_{i}\right)=-p_{i}logy_{i}$$

(20) $$L_{cs}=\sum_{i=1}^{N+M}E_{\eta^{\prime },\eta }{∥p_{i}^{\prime }-p_{i}∥}_{2}^{2}$$

During the later stages of training, category-adaptive pseudo-labels are incorporated. These pseudo-labels and their corresponding confidence scores are derived from the predictions of the teacher network on unlabeled data $x_{i}$(i=N+1,N+2,…,N+M). The pseudo-label loss $L_{pl}$ is then computed by combining these results with the predictions from the student network, as illustrated in Equation (17).

Consequently, the overall optimization objective of the entire framework can be formulated as

(21) $$L=L_{sup}+\lambda L_{cs}+\beta L_{scl}+\zeta L_{pl}$$

where λ represents an incrementally weighted factor, β denotes the hyperparameter for the labeled contrastive loss, and ζ signifies the hyperparameter for the pseudo-label loss. The ultimate objective is to minimize the loss function *L* by updating the student network’s parameters through gradient descent.

(22) $$\lambda \left(t\right)=1\cdot e^{-5\cdot {\left(1-\frac{t}{T}\right)}^{2}}$$

where λ(t) represents the Gaussian ramp-up curve that controls the weight, *t* denotes the current training iteration, and *T* is the ramp-up value. During the initial *T* training iterations, the function value gradually increases from 0 to 1. Subsequently, the value of λ is fixed at 1 for the remaining training process. This design ensures that, during the early stages of network training, when the consistency target of unlabeled data remains unreliable, the training loss will not be dominated by unsupervised loss.

The training algorithm of the semi-supervised medical image classification model based on supervised contrastive learning and class-adaptive pseudo-labels is shown in Algorithm 1.
**Algorithm 1:** Contrastive Learning and Class-Adaptive Pseudo-Labeling for Semi-Supervised Medical Image Classification
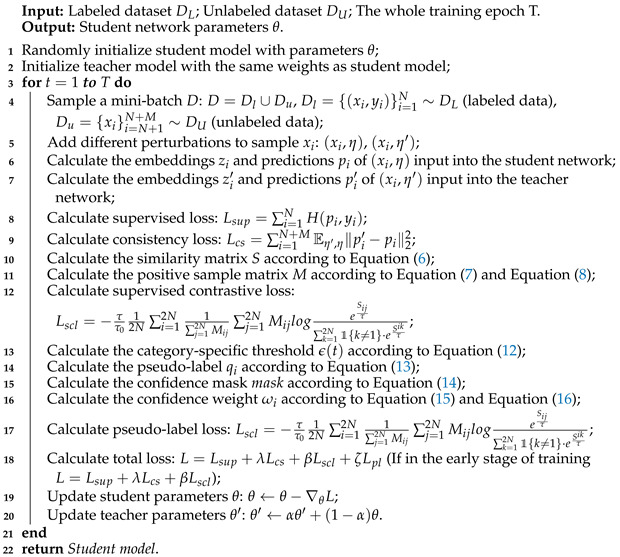


## 4. Experiments and Results

We evaluated our proposed semi-supervised learning approach for its application in dermoscopic image-based skin lesion classification (single-label) and chest X-ray image-based thoracic disease diagnosis (multi-label).

### 4.1. Datasets

#### 4.1.1. ISIC2018 Dataset

**Figure 5:**
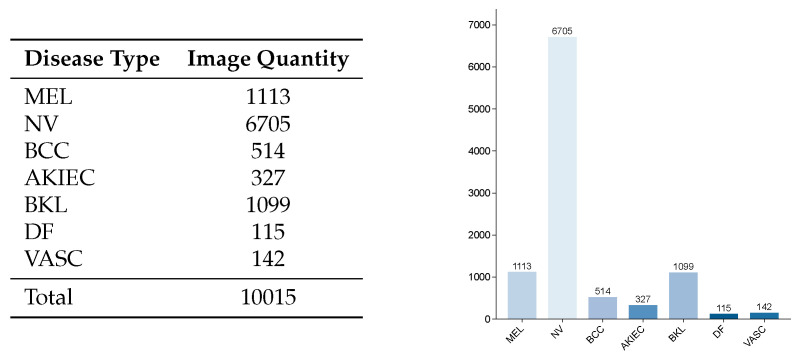
Distribution of categories in the ISIC2018 dataset.

We conducted skin lesion classification on the ISIC2018 dataset [36,37]. The ISIC2018 Task 3 dataset for skin lesion analysis, released by the International Skin Imaging Collaboration (ISIC) in 2018, comprises 12,500 dermoscopic images of skin lesions. This dataset includes 10,015 training images, 1512 test images, and 193 validation images. The training set contains seven disease categories, with each image having a resolution of 600 × 450 pixels. Specifically, the 10015 training images consist of 1113 melanoma (MEL) cases, 6705 melanocytic nevus (NV) cases, 514 basal cell carcinoma (BCC) cases, 327 actinic keratosis (AKIEC) cases, 1099 benign keratosis (BKL) cases, 115 dermatofibroma (DF) cases, and 142 vascular lesions (VASC). This constitutes a single-label imbalanced dataset, with the distribution of different lesion types illustrated in Figure 5.

All the images were resized to 224 × 224 pixels. To leverage pretrained models, we normalized each image from both datasets using statistical parameters derived from the ImageNet dataset [38]. For fair comparison and in accordance with prior work [17], we randomly partitioned the entire dataset into 70% for training, 10% for validation, and 20% for testing. Our network architecture employed DenseNet121 [39] pretrained on ImageNet [40] as the backbone.

#### 4.1.2. Chest X-Ray14 Dataset

**Figure 6:**
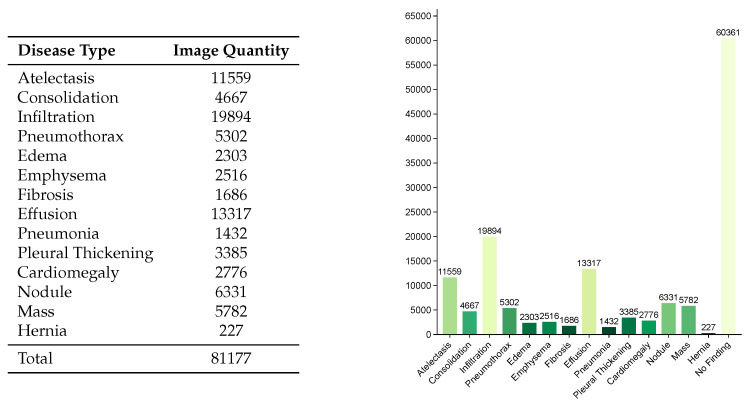
Distribution of categories in the Chest X-ray14 dataset.

We conducted thoracic disease diagnosis using the Chest X-ray14 dataset [41]. The Chest X-ray14 dataset, collected between 1992 and 2015, comprises 112,120 frontal-view X-ray images from 30,805 unique patients, along with image labels for 14 disease categories (each image may have multiple labels) extracted from corresponding radiology reports through natural language processing techniques. The images have a resolution of 1024 × 1024 pixels. The 14 diagnostic labels include Atelectasis, Consolidation, Infiltration, Pneumothorax, Edema, Emphysema, Fibrosis, Effusion, Pneumonia, Pleural Thickening, Cardiomegaly, Nodule, Mass, and Hernia. The distribution of different disease types is illustrated in Figure 6.

All the images were resized to a resolution of 224 × 224 pixels. To leverage the pretrained model, we normalized each image from both datasets using statistical parameters derived from the ImageNet dataset [38]. The official data partitioning protocol was adopted, allocating 70% of the samples for training, 10% for validation, and 20% for testing, with strict patient-wise separation ensuring no data leakage across the splits. Given the substantially larger scale of this dataset compared to ISIC2018, we implemented a deeper network architecture, specifically employing DenseNet169 [39] pretrained on ImageNet [40] as our backbone network.

#### 4.1.3. Evaluation Metric

We selected six evaluation metrics for assessment on the ISIC2018 dataset, including AUC (Area Under the Curve), sensitivity, specificity, accuracy, F1-score, and precision. For the Chest X-ray14 dataset, following previous work by [42], we adopted AUC as the evaluation metric.

### 4.2. Implementation Details

The experiments were conducted using Python 3.8 as the programming language and PyTorch 1.7.0 as the framework for the CLCP-MT model. To ensure a fair comparison, all the trials were performed on an NVIDIA RTX 3090 GPU with 24 GB of video memory (Santa Clara, CA, USA).

The experimental setup on the ISIC2018 dataset and studies conducted on the Chest X-ray14 dataset involved the following: All the configurations used DenseNet121 as the backbone. Each batch contained 48 samples: 12 labeled and 36 unlabeled. The models were trained for 100 epochs with a ramp-up period set to 30.

For the Chest X-ray14 dataset, we used DenseNet169 as the backbone. Each batch contained 48 samples: 12 labeled and 36 unlabeled. Training lasted for 20 epochs with a ramp-up period of 10.

The learning rate was set to 1 × 10^−4^ and decayed exponentially by 0.9 per epoch. We trained with the Adam optimizer and used an EMA decay of 0.99. Consistent with numerous SSL algorithms [17,22], we applied various perturbations to the input unlabeled images, including random cropping, flipping, color jittering, and blurring.

### 4.3. Comparison with the State-of-the-Art Methods

To validate the advancement of the CLCP-MT framework, we conducted comparative experiments with current mainstream semi-supervised learning approaches under identical experimental conditions. During the training process, all the methods used the same training configurations. These included data partitioning, preprocessing, input perturbations, learning rate schedulers, and optimizers. On the ISIC2018 dataset, all the methods used a pretrained DenseNet121 as the backbone. On the Chest X-ray14 dataset, all the methods used a pretrained DenseNet169 as the backbone.

#### 4.3.1. Results on ISIC2018 Dataset

On the ISIC2018 dataset, we conducted a comprehensive comparison between our proposed method and several state-of-the-art approaches, including consistency-based methods (mean teacher and SRC-MT), a pseudo-labeling-based method (NM), a hybrid approach combining consistency and pseudo-labeling (FixMatch), and a curriculum learning-based method (FlexMatch). The mean teacher model enforces prediction consistency between the student network and teacher network through consistency loss. The teacher’s parameters are updated as the exponential moving average of the student’s parameters. Building upon mean teacher, SRC-MT incorporates SRC loss to model relational information among different samples for effective utilization of unlabeled data. The NM method functions as a pseudo-label estimator that propagates labels based on neighboring samples of unlabeled data. FixMatch generates pseudo-labels using a fixed threshold. It then computes cross-entropy loss between the pseudo-labels and strongly augmented predictions of the same sample. FlexMatch enhances FixMatch by replacing the fixed threshold with a dynamic threshold mechanism.

As shown in Table 1, we conducted experiments using 20% labeled data and 80% unlabeled data. The upper bound represents the fully supervised model trained with 100% (7000) labeled data, serving as the performance ceiling. The baseline denotes the fully supervised model trained with only 20% (1400) labeled data. Our proposed method outperforms other state-of-the-art approaches across all the metrics except sensitivity. Compared to the mean teacher model, which also enforces prediction consistency between two models, the CLCP-MT model achieves improvements of 1.44% in AUC, 0.28% in specificity, 0.81% in accuracy, 4.8% in F1-score, and 8.17% in precision. Relative to the FixMatch method that similarly constrains pseudo-labels with predictions from another model, CLCP-MT demonstrates enhancements of 0.87% in AUC, 0.77% in specificity, 0.04% in accuracy, 1.91% in F1-score, and 3.95% in precision. When compared to FlexMatch, which also employs dynamic thresholds, our CLCP-MT model shows superior performance with gains of 1.03% in AUC, 0.89% in specificity, 0.01% in accuracy, 2.01% in F1-score, and 3.86% in precision.

The experimental results demonstrate that all the methodologies outperform the baseline, indicating that unlabeled data can benefit the model. The mean teacher model enforces consistency between the predictions of the student and teacher networks, while the SRC-MT model incorporates a sample relation consistency paradigm on top of the mean teacher framework, leading to improvements in AUC, sensitivity, accuracy, F1-score, and precision, albeit with a 0.11% decline in specificity. This suggests that SRC-MT effectively leverages unlabeled sample information, albeit at the cost of increased false positives among negative samples with similar features. Compared to the mean teacher model, the FixMatch model exhibits superior performance across all the evaluation metrics except specificity, indicating its enhanced capability in detecting positive samples. The FlexMatch model employs a dynamic thresholding mechanism for pseudo-label generation but underperforms relative to FixMatch, suggesting its limited applicability to imbalanced medical imaging datasets. The NM model propagates labels based on neighboring unlabeled samples, achieving higher sensitivity than the other methods, which implies fewer missed diagnoses of diseases. Our proposed method surpasses all the comparative approaches in five out of six evaluation metrics, with only sensitivity being marginally lower than that of the NM model, demonstrating its superior effectiveness in utilizing unlabeled data.

#### 4.3.2. Results on Chest X-Ray14 Dataset

On the Chest X-ray14 dataset, our approach was benchmarked against the consistency-based mean teacher model and the SRC-MT model, both of which were previously described in Section 4.3.1.

As illustrated in Table 2, our experimental setup comprised 20% labeled data and 80% unlabeled data. The upper bound represents the fully supervised model utilizing 100% (78,468) labeled data, serving as the performance ceiling for this experiment. The baseline constitutes the fully supervised model trained solely on 20% (15,694) labeled data, establishing our experimental starting point. Compared to the baseline, the CLCP-MT model demonstrated a 2.64% improvement in average AUC values. Relative to the upper bound, the CLCP-MT model exhibited a 7.73% reduction in average AUC performance. When benchmarked against the MT model, the CLCP-MT architecture achieved AUC improvements across eight categories, with the Hernia class (representing only 0.2% of the total dataset as an extreme minority) showing a 2.09% higher average AUC. Similarly, the CLCP-MT model outperformed the SRC-MT model in eight categories, delivering a 1.36% enhancement in mean AUC scores.

Compared to the baseline, the CLCP-MT model demonstrates improved average AUC values, indicating its effective utilization of additional discriminative information derived from unlabeled data. However, when benchmarked against the upper bound, our approach still exhibits potential for further enhancement in leveraging unlabeled data. The CLCP-MT model achieves the highest average AUC values among the comparative methods, outperforming both mean teacher and SCR-MT. Notably, it exhibits superior AUC performance in classifying the Hernia, Pneumonia, and Fibrosis categories—three underrepresented classes in the Chest X-ray14 dataset, comprising merely 0.2%, 1.28%, and 1.5% of the total dataset, respectively. This result underscores our model’s enhanced capability in recognizing tail-class data.

### 4.4. Ablation Study

#### 4.4.1. Different Percentages of Labeled Data

We conducted ablation experiments on the ISIC2018 dataset under varying percentages of labeled training data. The upper bound represents the fully supervised model trained with 100% labeled data, while the baseline constitutes the fully supervised model trained exclusively on labeled data. Four label proportions (5%, 10%, 20%, and 30%) were selected for comparative analysis against both the baseline under different labeled data ratios and the upper bound.

As demonstrated in Table 3, the CLCP-MT model consistently outperforms the baseline across all four labeled data proportions (5%, 10%, 20%, and 30%). When utilizing 5% labeled data, the CLCP-MT model achieves improvements of 2.08% in AUC, 0.32% in sensitivity, 2.88% in specificity, 6.05% in accuracy, 12.12% in F1-score, and 15.06% in precision compared to the baseline using only 5% labeled data. With 20% labeled data, the model exhibits enhancements of 3.88% in AUC, 3.2% in sensitivity, 1.18% in specificity, 3.58% in accuracy, 10.38% in F1-score, and 14.46% in precision relative to the corresponding baseline. Furthermore, when comparing the performance between 20% and 30% labeled data, the CLCP-MT model shows additional gains of 0.46% in AUC, 6.05% in sensitivity, 0.86% in specificity, 0.08% in accuracy, and 0.85% in F1-score. At the 30% labeled data level, the model’s AUC of 92.71% approaches the upper bound of 94.77% with a marginal gap of 2.06%, while its accuracy of 93.76% nears the upper bound of 95.29% with only a 1.53% difference. However, the F1-score still lags behind by 8.4%, indicating potential for further improvement in positive class identification.

Under four distinct label proportions (5%, 10%, 20%, and 30%), the CLCP-MT model leveraging unlabeled data demonstrated statistically significant superiority over the baseline that solely utilized labeled data across all the evaluation metrics. This performance advantage remained consistent with increasing labeled data quantities. Specifically, when employing only 5% labeled data, our method achieved a 12.12% higher F1-score than the baseline through the utilization of unlabeled data, substantiating the model’s capability to extract valuable information from unlabeled datasets. While the model performance exhibited progressive improvement with additional labeled data, the enhancement became statistically insignificant when increasing from 20% to 30% labeled data. This observation indicates that augmenting labeled data yields diminishing returns: it substantially benefits model performance under data-scarce conditions but approaches a performance plateau as labeled data quantities increase.

Table 4 presents a comparative analysis of the mean AUC values between the CLCP-MT model and the MT model across varying proportions of labeled data in the Chest X-ray14 dataset. The CLCP-MT model consistently demonstrates superior performance under all the labeled data ratios. Specifically, with 2% labeled data, the CLCP-MT model achieves a 1.04% higher mean AUC value compared to the MT approach. When utilizing 20% labeled data, our method exhibits a more substantial improvement of 2.09% in mean AUC over the MT baseline. The progressive enhancement in the CLCP-MT model’s mean AUC with increasing labeled data quantities substantiates the positive impact of labeled data on model performance.

#### 4.4.2. Effect of Weight Coefficient Results of Two Unlabeled Losses

We investigated the impact of varying hyperparameters β and ζ in Equation (21). Here, β denotes the weight of the labeled data-contrastive loss, while ζ represents the weight of the pseudo-label loss. Both β and ζ were selected from the range [0, 1]. Specifically, we examined the values β,ζ∈{0,0.1,0.25,0.5,1.0}. The ablation study of these weight hyperparameters is presented in Table 5. The configuration β=0 and ζ=0 indicates that the model exclusively employs supervised loss and consistency loss.

In comparison to the baseline scenario with β=0 and ζ=0, the incorporation of labeled data-contrastive loss and pseudo-label loss yields statistically significant improvements across all the evaluation metrics, including AUC, accuracy, and F1-score. The AUC metric demonstrates a maximum enhancement from 90.81% to 92.25%, while the F1-score shows a more substantial increase from 55.87% to 61.82%, indicating a positive impact of these loss functions on the model’s classification performance. Within the parameter range of β,ζ∈[0,1], the model exhibits greater sensitivity to variations in β. The optimal AUC value of 92.25% is achieved at β=0.5 and ζ=0.25, with the accuracy reaching a suboptimal value of 93.68%. Beyond this parameter configuration, further increases in either weight parameter result in diminishing returns or marginal performance degradation.

The incorporation of contrastive learning and pseudo-labeling methods can effectively enhance the overall model performance. Appropriately increasing the β value improves the model’s capability to capture positive samples, whereas excessive augmentation of β may lead to reduced sensitivity. We systematically selected parameters that optimize the model’s discriminative ability between positive and negative samples, ultimately employing β=0.5 and ζ=0.25 for subsequent experimental validation.

#### 4.4.3. Effect of Different Components

We conducted ablation studies using 20% (1400) labeled data and 80% (5600) unlabeled data from the training set. Our proposed CLCP-MT model consists of three components: a consistency regularization method, a semantic discrimination enhancement module, and a category-adaptive pseudo-label regulation module. To further investigate the contribution of each component to the overall model performance, ablation experiments were carried out, as shown in Table 6.

The experimental results demonstrate that incorporating the consistency regularization method into the baseline model led to improvements of 2.44%, 3.74%, 0.90%, 2.77%, 5.58%, and 6.28% in AUC, sensitivity, specificity, accuracy, F1-score, and precision, respectively. Building upon the consistency regularization method, the addition of the SDE module further enhanced AUC, accuracy, F1-score, and precision, although sensitivity decreased by 0.90%. When the CAPR module was integrated on top of the consistency regularization method, AUC, sensitivity, F1-score, and precision increased by 1.83%, 0.06%, 0.05%, and 0.05%, respectively, while specificity and accuracy decreased by 0.79% and 0.22%. Finally, with both the SDE and CAPR modules added alongside the consistency regularization method, all the evaluation metrics showed improvements.

The aforementioned results demonstrate that the consistency regularization method significantly enhances the model’s overall classification performance by enforcing output-level consistency between the student and teacher networks. While the SDE module facilitates deeper exploitation of discriminative information from labeled data, it may inadvertently push scarce positive samples toward negative regions in the highly class-imbalanced ISIC2018 dataset, consequently reducing sensitivity. The CAPR module introduces additional supervisory signals for unlabeled data, thereby strengthening the model’s discriminative capability overall. However, the inherent noise in pseudo-labels can adversely affect precision, leading to a degradation in the F1-score.

## 5. Conclusions

In this paper, we propose a semi-supervised medical image classification method based on contrastive learning and category-adaptive pseudo-labeling. The approach leverages supervised contrastive loss to minimize intra-class distances while maximizing inter-class distances, thereby fully utilizing limited labeled data and effectively extracting structural information from annotated samples. Additionally, we developed a category-adaptive pseudo-label generation mechanism that dynamically assigns pseudo-labels to unlabeled samples based on class-specific thresholds. This innovative method mitigates the dominant influence of head classes and significantly improves the recognition rate of tail categories. Our methodology demonstrates enhanced performance in semi-supervised medical image classification. Future work will focus on addressing challenges arising from class imbalance through advanced semi-supervised classification techniques.

## Figures and Tables

**Table 1:** Comparison with state-of-the-art methods on ISIC2018 dataset (%).

Method	Labeled	Unlabeled	Metrics					
			**AUC**	**Sensitivity**	**Specificity**	**Accuracy**	**F**1	**Precision**
Upper Bound	100	0	94.77	71.85	95.47	95.29	69.92	68.09
Baseline	20	0	88.37	61.90	91.27	90.10	50.29	42.35
Mean Teacher [21]	20	80	90.81	65.64	92.17	92.87	55.87	48.63
SRC-MT [17]	20	80	91.16	65.86	92.06	93.17	58.45	52.54
FixMatch [25]	20	80	91.38	66.16	91.68	93.64	58.76	52.85
FlexMatch [26]	20	80	91.22	65.78	91.56	93.67	58.66	52.94
NM [43]	20	80	89.76	**67.14**	87.32	89.87	49.34	39.02
CLCP-MT (ours)	20	80	**92.25**	65.10	**92.45**	**93.68**	**60.67**	**56.80**

**Table 2:** Comparison of AUC values for different semi-supervised methods on the Chest X-ray14 dataset (%).

Disease Category	Metrics				
	**Upper Bound**	**Baseline**	**MT [21]**	**SRC-MT [17]**	**CLCP-MT (Ours)**
Atelectasis	73.80	68.26	66.57	64.75	**69.97**
Consolidation	77.11	57.96	**68.93**	64.81	64.79
Infiltration	50.33	52.79	**52.79**	50.57	50.42
Pneumothorax	84.78	68.66	64.41	70.71	**73.94**
Edema	86.81	80.92	**82.47**	81.29	80.22
Emphysema	90.71	71.79	75.51	76.54	**79.01**
Fibrosis	76.49	67.36	68.14	68.35	**73.78**
Effusion	86.67	81.80	**83.61**	81.05	82.67
Pneumonia	74.55	50.52	56.03	54.10	**56.59**
Pleural Thickening	71.61	63.50	64.25	63.18	**69.26**
Cardiomegaly	87.62	84.10	**84.61**	82.23	78.88
Nodule	66.42	60.05	60.27	**62.36**	61.84
Mass	79.96	59.09	66.94	**70.42**	66.35
Hernia	92.67	87.63	67.56	81.89	**83.60**
Average AUC	78.54	68.17	68.72	69.45	**70.81**

**Table 3:** Classification results on the ISIC2018 dataset with different proportions of labeled data (%).

Method	Labeled	Unlabeled	Metrics					
			**AUC**	**Sensitivity**	**Specificity**	**Accuracy**	**F1**	**Precision**
Upper Bound	100	0	94.77	71.85	95.47	95.29	69.92	68.09
Baseline	5	0	81.11	55.60	87.01	83.24	34.82	25.37
CLCP-MT (ours)	5	95	**83.19**	**55.92**	**89.89**	**89.29**	**46.94**	**40.43**
Baseline	10	0	87.06	59.89	89.56	87.98	44.74	35.70
CLCP-MT (ours)	10	90	**89.69**	**64.84**	**91.08**	**92.31**	**57.59**	**51.80**
Baseline	20	0	88.37	61.90	91.27	90.10	50.29	42.35
CLCP-MT (ours)	20	80	**92.25**	**65.10**	**92.45**	**93.68**	**60.67**	**56.81**
Baseline	30	0	89.56	66.60	91.98	90.24	55.40	47.42
CLCP-MT (ours)	30	70	**92.71**	**71.15**	**93.31**	**93.76**	**61.52**	**54.19**

**Table 4:** Classification results on the Chest X-ray14 dataset with different proportions of labeled data (%).

Labeled Percentage	2%	5%	10%	15%	20%
MT [21]	59.50	64.49	65.06	68.12	68.72
CLCP-MT (ours)	**60.46**	**65.30**	**66.32**	**69.14**	**70.81**

**Table 5:** Comparison of results on ISIC2018 dataset for two different weighting parameters for loss (%).

Method	Loss Weight		Metrics					
	β	ζ	**AUC**	**Sensitivity**	**Specificity**	**Accuracy**	**F1**	**Precision**
CLCP-MT (ours)	0	0	90.81	65.64	92.17	92.87	55.87	48.68
CLCP-MT (ours)	0.1	0.1	91.73	66.04	91.13	93.47	59.37	53.92
CLCP-MT (ours)	0.25	0.1	91.71	**67.11**	92.26	93.65	61.76	57.20
CLCP-MT (ours)	0.25	0.25	91.73	67.04	92.24	93.66	**61.82**	**57.35**
CLCP-MT (ours)	0.5	0.25	**92.25**	65.10	92.45	93.68	60.67	56.81
CLCP-MT (ours)	0.5	0.5	92.22	64.35	92.43	93.65	60.07	56.32
CLCP-MT (ours)	1.0	0.5	91.63	64.23	92.52	**93.73**	59.53	55.48
CLCP-MT (ours)	1.0	1.0	91.62	64.17	92.54	**93.73**	59.51	55.48

**Table 6:** Results of ablation experiments for each module on the ISIC2018 dataset (%).

Consistency Regularization	**SDE**	**CAPR**	**AUC**	**Sensitivity**	**Specificity**	**Accuracy**	**F1**	**Precision**
×	×	×	88.37	61.90	91.27	90.10	50.29	42.35
✓	×	×	90.81	65.64	92.17	92.87	55.87	48.63
✓	✓	×	91.60	64.74	92.23	93.65	**61.62**	**58.78**
✓	×	✓	92.64	**65.70**	91.38	92.65	55.92	48.68
✓	✓	✓	**92.25**	65.10	**92.45**	**93.68**	60.67	56.80

## Data Availability

The experimental results in this study are based on the publicly available ISIC2018 and Chest X-ray14 datasets, which can be accessed at https://challenge.isic-archive.com/data (accessed on 20 August 2025) and https://paperswithcode.com/dataset/chestx-ray14 (accessed on 20 August 2025), respectively.
